# Supplementary material for: Construction and Characterization of Polyolefin Elastomer Blends with Chemically Modified Hydrocarbon Resin as a Photovoltaic Module Encapsulant
Source: Polymers (Basel). 2022 Oct 31;14(21):4620. doi: 10.3390/polym14214620 (PMC9658822; doi:10.3390/polym14214620)
Supplement: Supplementary file 1 [file polymers-14-04620-s001.zip › polymers-1986928-SI.pdf]

Supporting Information for;

Construction of Adhesion-enhanced Polyolefin Elastomer Encapsulant using Chemically Modified Hydrocarbon Resin for Construction and Characterization of Polyolefin Elastomer Blends with Chemically Modified Hydrocarbon Resin as a Photovoltaic Module Encapsulant

Jin Hwan Park and Seok-Ho Hwang *

Materials Chemistry and Engineering Laboratory, Department of Polymer Science and Engineering, Dankook University, Yongin 16890, Gyeonggi-do, Korea; shssh123@gmail.com

* Correspondence: bach@dankook.ac.kr; Tel.: +82-31-8005-3588

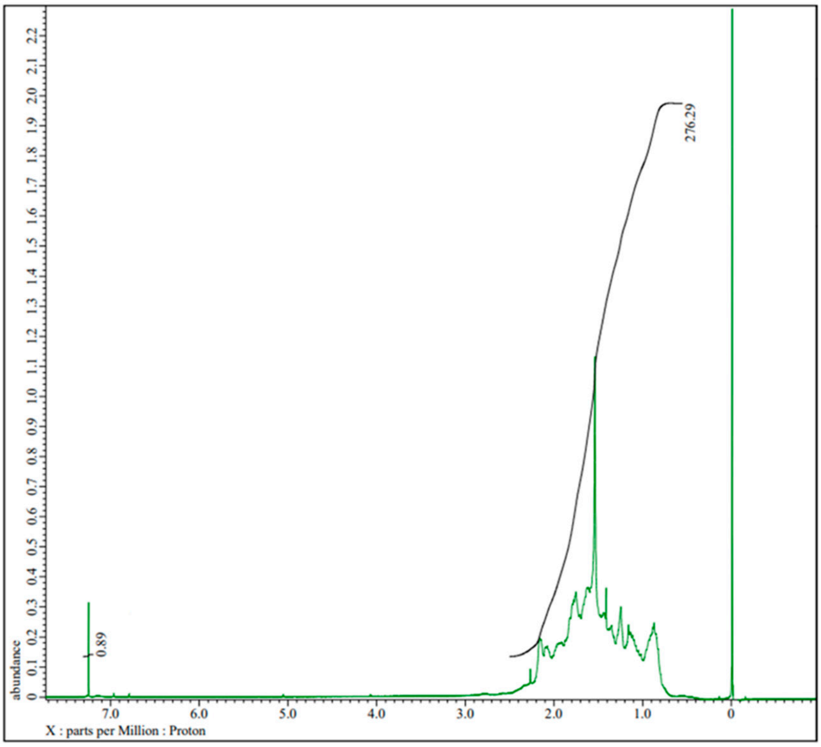

(A)

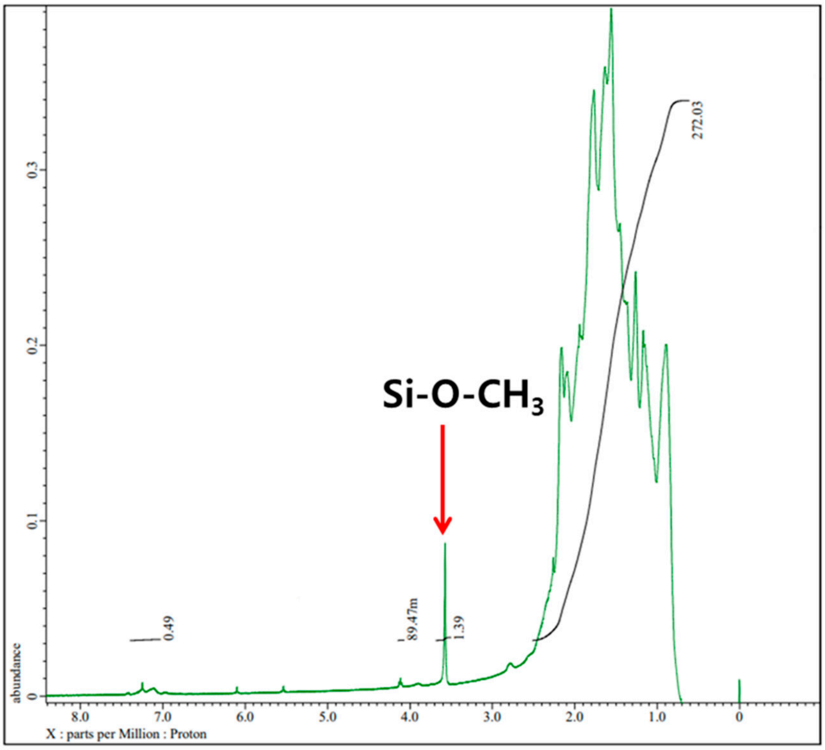

(B)

Figure S1. ^{1}H NMR spectra of the neat HCR (A) and *m*-HCR (B).

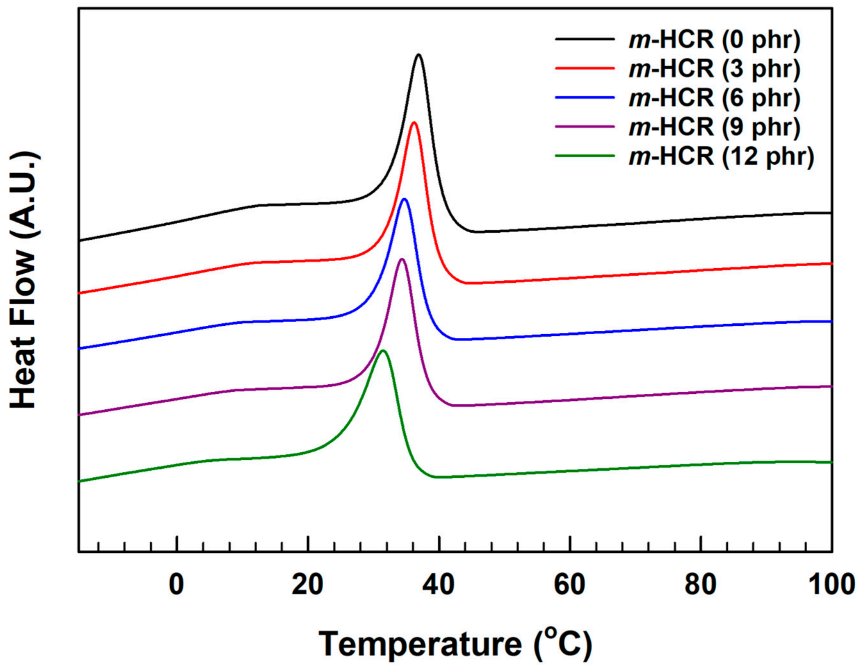

(A)

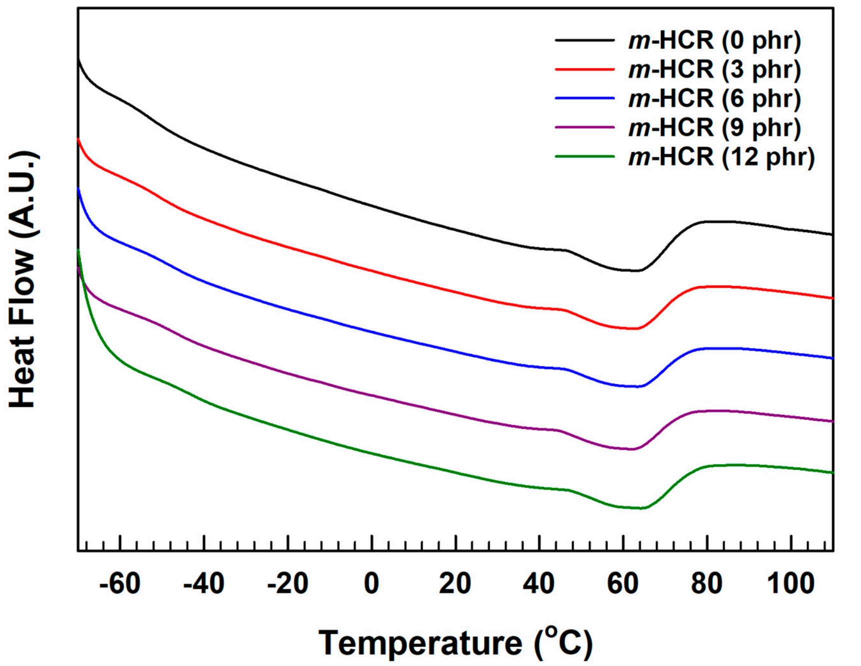

(B)

<Continued>

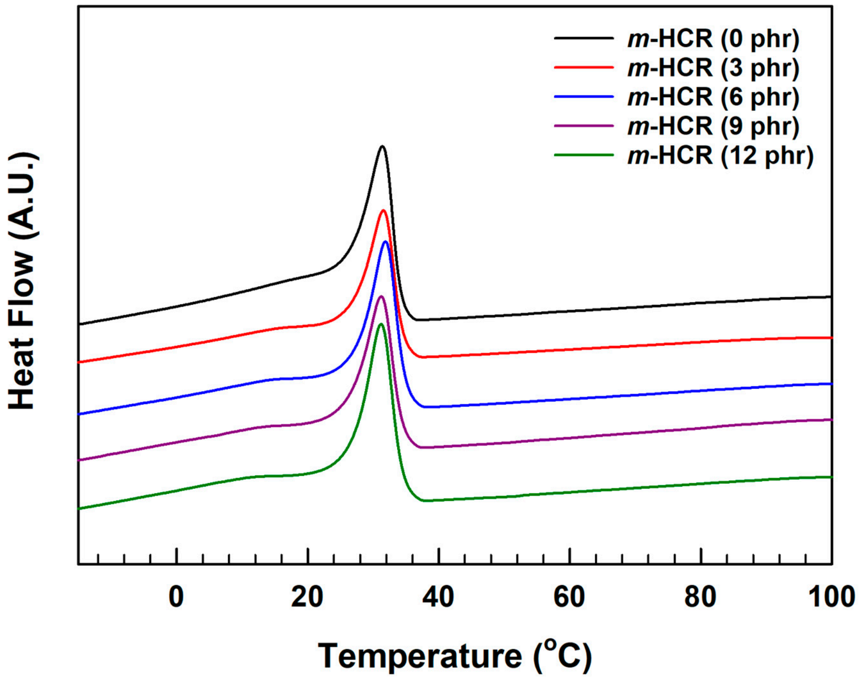

(C)

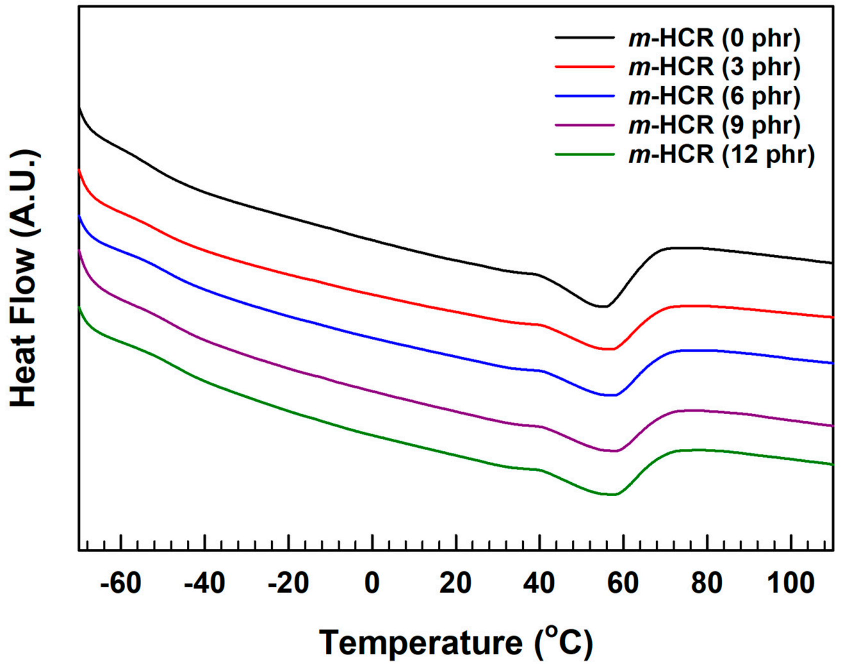

(D)

Figure S2. DSC thermograms of neat POE and POE blends with *m*-HCR: (A) cooling scan, (B) 2nd heating scans for uncrosslinked samples and (C) cooling scan, (B) 2nd heating scan for crosslinked samples

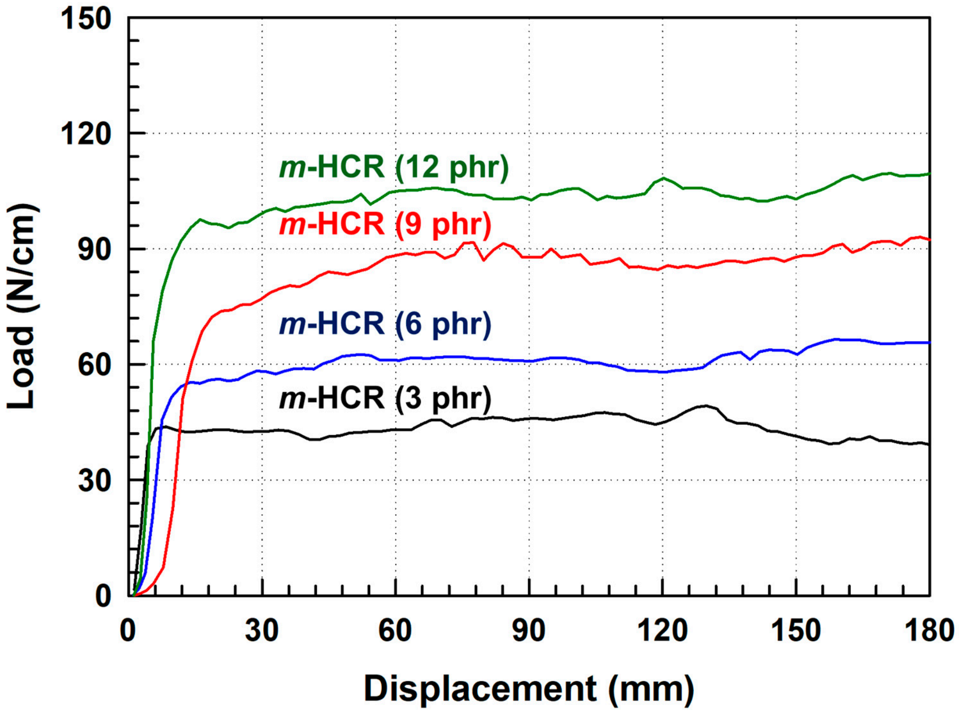

Figure S3. 180° Peel test profiles for POE blends with *m*-HCR.
